# Current status of vaccine clinical trials registered on China's Drug Trial Registration and information disclosure platform, 2013–2024

**DOI:** 10.3389/fpubh.2026.1744834

**Published:** 2026-04-07

**Authors:** Sumei Zhong, Zhiqiang Lin, Junrong Li, Fangqin Xie, Dongjuan Zhang

**Affiliations:** 1 Fujian Provincial Center for Disease Control and Prevention, Fuzhou, Fujian, China; 2 Fujian Provincial Center for Disease Control and Prevention, Fujian Provincial Key Laboratory of Zoonosis Research, Fuzhou, Fujian, China

**Keywords:** China, clinical trials, epidemiology, public health, regulatory trends, vaccine

## Abstract

**Background:**

Vaccines are critical tools in global infectious disease prevention, especially amid emerging threats such as COVID-19 and HPV-related cancers. In China, vaccine development has gained momentum due to increasing public health demands and supportive regulatory reforms. However, comprehensive analyses of the broader landscape of vaccine clinical trials remain limited. This study aims to provide a systematic overview of vaccine clinical trials registered in China from 2013 to 2024, examining trends in trial design, phase distribution, vaccine types, and geographic distribution.

**Methods:**

We retrieved data from the Drug Trial Registration and Information Publication Platform using keywords including “vaccine,” “BCG,” and “toxoid.” After deduplication and exclusion of non-vaccine entries, 620 eligible vaccine clinical trials were included. Data on trial design, phase, recruitment status, participant demographics, vaccine type, and institutional affiliations. The data were extracted and statistically analyzed by Microsoft Excel 2021, IBM SPSS Statistics version 21andArcGIS 10.8.1, respectively.

**Results:**

Among the 620 trials, the majority were parallel-group (84.84%), randomized (81.94%), and double-blind (67.58%). Phase I and III trials accounted for the largest proportions (30.27% and 35.75%, respectively). Influenza (17.1%), HPV (13.4%), and pneumococcal (9.8%) vaccines were the most frequently studied. Most trials were led by CDCs (92.3%) and conducted in eastern provinces, with Jiangsu, Henan, and Guangxi accounting for over half of all trials. The number of registered trials increased steadily from 2013 to 2019, peaking in 2021 and 2023.

**Conclusions:**

China has seen consistent growth in vaccine clinical trial activity, supported by regulatory enhancements and public health priorities. While trial volume and geographic spread have expanded, opportunities remain to increase multi-center collaboration, insurance coverage, and diversity in trial phases and vaccine types. These findings provide a foundation for evidence-based policy-making and strategic planning in China's vaccine development landscape.

## Introduction

Vaccines are among the most effective public health interventions for preventing infectious diseases and reducing mortality worldwide (1–3). With the continuous emergence of new infectious threats such as COVID-19, avian influenza, and HPV-related cancers, vaccine development has become a global research priority. Compared to other pharmaceuticals, vaccine clinical trials are often more complex, requiring large sample sizes, long-term follow-up, and rigorous safety evaluations (4, 5).

In China, one of the world's most populous countries, the demand for vaccines is enormous, driven by both national immunization programs and public health initiatives. Over the past decade, China has made significant investments in vaccine research and development, with a growing number of domestic pharmaceutical companies entering the vaccine market (6–8). The government has also implemented a series of regulatory reforms to support vaccine innovation. Since 2013, the Chinese government has mandated the registration of all vaccine clinical trials through the Drug Trial Registration and Information Publication Platform, aiming to enhance transparency and regulatory oversight (9, 10). The enactment of the Vaccine Management Law in 2019 further strengthened the legal framework for vaccine development, quality control, and post-marketing surveillance. These regulatory advancements have contributed to a steady increase in the number of registered vaccine trials, particularly in pediatric and endemic disease areas.

However, despite this progress, systematic analyses of the overall landscape of vaccine clinical trials in China remain limited. Existing studies often focus on specific vaccine types or specific populations, leaving a gap in understanding the broader trends across all vaccine trials, including trial phases, disease indications, geographic distribution, and design type (11, 12).

This study aims to fill that gap by providing a comprehensive overview of vaccine clinical trials registered in China from 2013 to 2024. By analyzing trial characteristics and trends, this research seeks to inform future vaccine development strategies and policy-making in China's evolving regulatory environment.

## Methods

### Data source

We investigate the current status of vaccine clinical trials registered in the Drug Trial Registration and Information Publication Platform (http://www.chinadrugtrials.org.cn/index.html) before December 31, 2024. As explicitly stipulated in the Announcement of the State Food and Drug Administration on the Information Platform for Drug Clinical Studies (No. 28, 2013), all entities that have obtained clinical trial approval documents from the China Food and Drug Administration (CFDA) and conduct clinical trials in China (including bioequivalence trials, pharmacokinetic (PK) trials, Phase I, II, III, and IV trials, etc.) shall log in to the information platform and complete clinical trial registration and information disclosure in accordance with relevant requirements (13). To ensure complete ascertainment of vaccine clinical trials, we systematically searched trial registries for records that contained any of the following keywords: “vaccine,” “BCG,” or “toxoid.” After deduplication, two reviewers independently screened the retrieved entries against predefined criteria. Studies were excluded if (a) essential registry fields were incomplete, or (b) the name included the search keyword but the investigational product was not, by mechanism of action and regulatory definition, a vaccine. For example, “Extracts from Rabbit Skin Inflamed by Vaccinia Virus for Injection” contains the word “vaccine” in its Chinese approved name, yet its pharmacological action is anti-inflammatory and unrelated to antigen-specific immunization; such agents were therefore excluded. The following trial characteristics were extracted: registration number, recruitment status, phase, date of first public disclosure, province of the leading site, number of participating centers, vaccine types, target indication, sex and age of participants, presence of a data monitoring committee (DMC), and whether insurance coverage was purchased for participants.

## Statistical analysis

Descriptive statistics were used to summarize the data. Categorical variables are presented as frequency (percentage). A simple regression model was fitted to examine the temporal trend in the number of registered trials, and the average annual growth rate was calculated from the slope. The chi-square test was used to compare trial design characteristics between the pre-implementation (2013–2018) and post-implementation (2019–2024) periods. All statistical tests were two-tailed, and *P* < 0.05 was considered statistically significant. The trial year was defined according to the date of first public disclosure. All analyses were performed with Microsoft Excel 2021 and IBM SPSS Statistics version 21 on a personal computer. The source of the map of China is the Alibaba Cloud Data Visualization Platform (https://datav.aliyun.com/portal/school/atlas/area_selector) with the map review number GS2024 (1,158). The choropleth map was produced using ArcGIS 10.8.1.

## Results

### 1 General characteristics of the trials

A total of 27,972 drug clinical trials were registered on the platform as of December 31, 2024. Using the keywords “vaccine,” “BCG,” and “toxoid,” we retrieved 614, 7, and 2 entries, respectively. After removing two duplicates and one non-vaccine entry, 620 vaccine clinical trial records were finally included, accounting for 2.22% of all trials registered during the same period. Table 1 summarizes the general characteristics of 620 vaccine clinical trials conducted in China. In terms of trial design, most trials adopted a parallel-group design, randomization, and a double-blind design. The vast majority of these trials were preventive vaccine clinical trials targeting the general population. Most trials included both male and female participants; among the remaining trials, the overwhelming majority focused on female participants, with relatively few vaccine clinical trials targeting male participants. In addition, most trials neither established a DMC nor provided insurance coverage. But between 2013 and 2024, the insurance purchase rate for clinical trials showed a significant upward trend, and this trend can be clearly divided into three phases. A simple regression model revealed that the overall average annual growth rate of the number of insurance purchase through 2013 to 2024 was 28.4% (*P* < 0.05). During the early phase (2013–2018), the purchase rate remained at a low level, fluctuating between 0 and 14%. The transition phase (2019–2020) witnessed a significant surge in the purchase rate, jumping sharply from 23.19 to 47.06% (24/51) and achieving a phased breakthrough. In the mature phase (2021–2024), the purchase rate stabilized at a high level and continued to rise, climbing gradually from 44.44 to 76.12% (51/67) in 2024—representing the highest rate recorded over the entire period.

**Table 1:** General characteristics of 620 vaccine clinical trials in China.

Items	*n* (%)
Design	
Single-arm trial	91 (14.68%)
Parallel-group	526 (84.84%)
15.6-7.8,-1.3242ptFactorial design	3 (0.48%)
Randomization	
Randomized	508 (81.94%)
15.6-7.8,-1.3242ptNon-randomized	112 (18.06%)
Blinding	
Single-blind	19 (3.06%)
Double-blind	419 (67.58%)
15.6-7.8,-1.3242ptOpen-label	182 (29.35%)
Healthy volunteers	
No	31 (5.00%)
15.6-7.8,-1.3242ptYes	589 (95.00%)
Gender	
Male	6 (0.97%)
Female	67 (10.81%)
15.6-7.8,-1.3242ptBoth	547 (88.23%)
DMC	
No	534 (86.13%)
15.6-7.8,-1.3242ptYes	86 (13.87%)
Insurance	
No	392 (63.23%)
15.6-7.8,-1.3242ptYes	228 (36.77%)
Trial range	
International	6 (0.97%)
15.6-7.8,-1.3242ptChina	614 (99.03%)
No.of centers	
Single-center trial	528 (85.16%)
Multiple-center trial	92 (14.84%)

### 2 Trial distribution by phase and year

**Figure 1:**
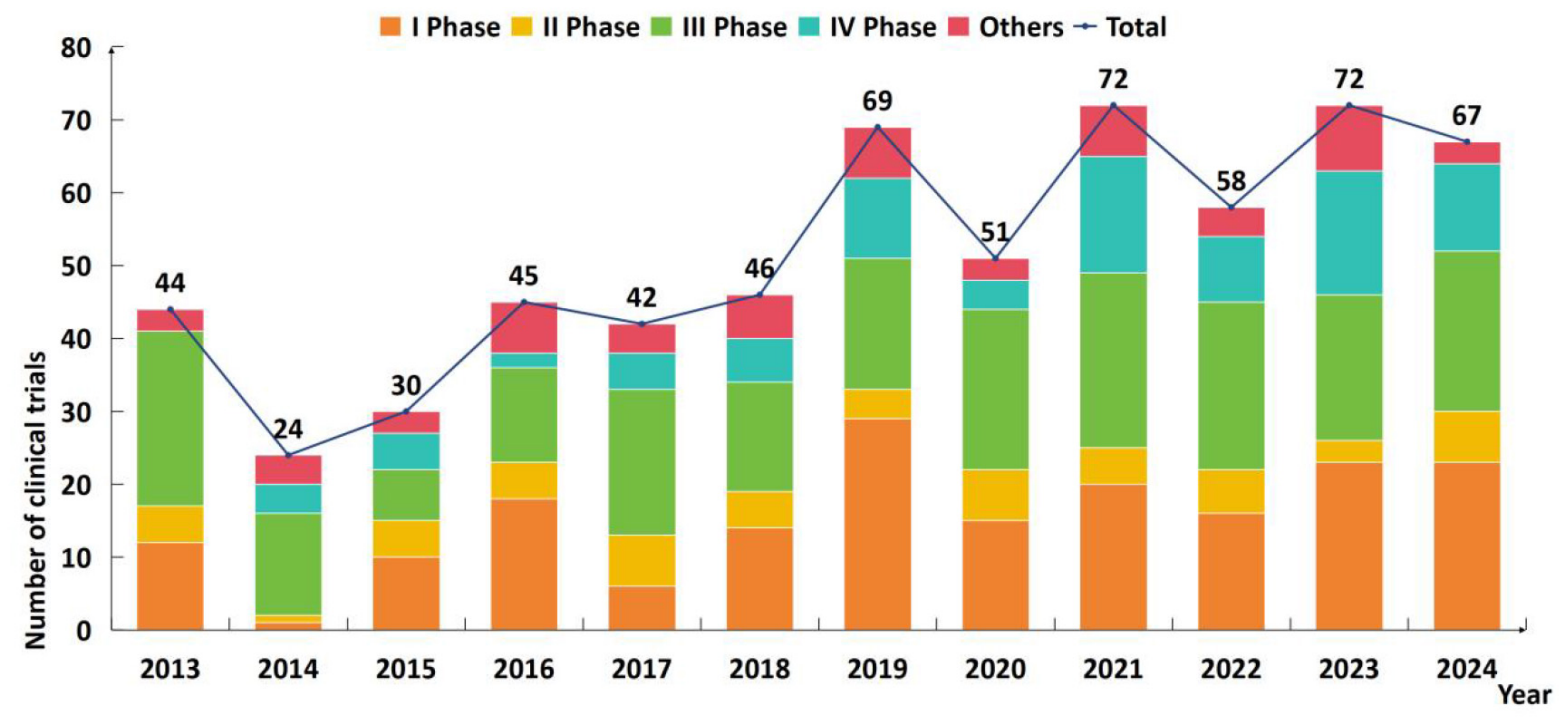
Number of vaccine clinical trials in China (the upper end of the column was marked as the total number).

By considering the first public disclosure date, the number and the phase of trials distributed in different years was shown in Figure 1. Phase I trials accounted for the highest proportion, reaching 30.27% (*n* = 187), followed by Phase III trials at 35.75% (*n* = 222). Phase IV trials represented 14.65% (*n* = 91), whereas Phase II and the “other” category each comprised 9.66 % (*n* = 60). When examined by year, 2019 marked the first time the annual count exceeded 60 (*n* = 69), representing a 47% increase over 2018. A first peak was observed in 2021 (*n* = 72), and an identical peak was again reached in 2023. The longitudinal trend from 2013 to 2019 demonstrated a consistent increase, rising from 44 to 69 trials. A simple regression model revealed that the overall average annual growth rate of the number of trials through 2013 to 2024 was 5.13% (*P* < 0.05). More recently, the period 2020–2023 exhibited fluctuation within the range of 51–72 trials. Collectively, the three most recent years (2022–2024) contributed 31.23% of the total.

### 3 Classification of vaccine clinical trials

**Figure 2:**
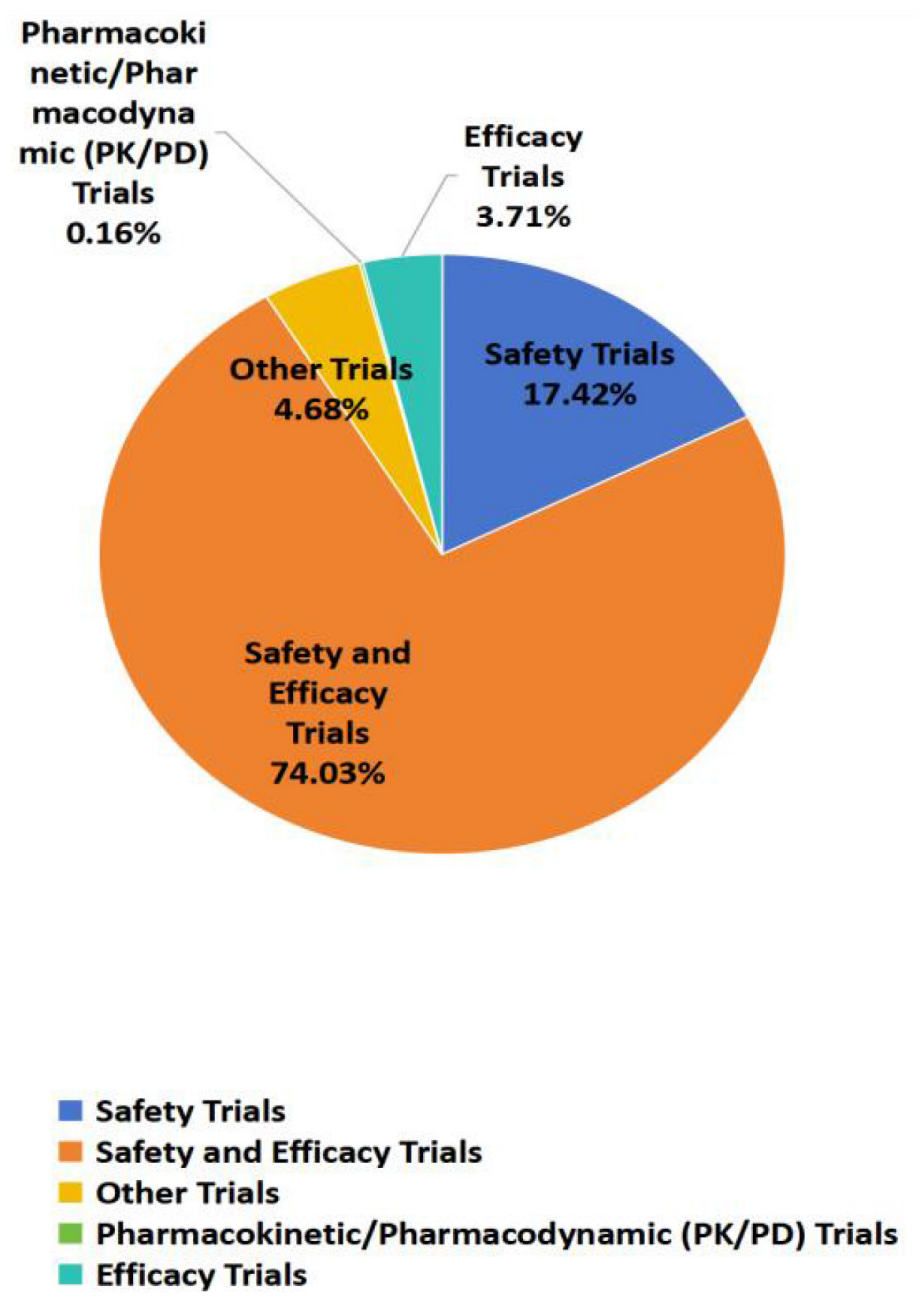
Classification of vaccine clinical trials in China.

Figure 2 shows the classification of vaccine clinical trials in China. Among vaccine clinical trials, the overwhelming majority (*n* = 459, 74.03%) were hybrid safety-and-efficacy studies. Stand-alone safety assessments accounted for 108 trials (17.42%), whereas pure efficacy trials comprised only 23 (3.71%). The remaining 29 studies (4.68%) were classified as“other”, encompassing narrowly focused objectives such as immunogenicity, lot-to-lot consistency, and persistence of immune response; each substratum represented 0.16% (1–3 trials). Pharmacokinetic/pharmacodynamic(PK/PD) evaluations were virtually absent (0.16%, *n* = 1). Only the clinical study on the protective mechanism of immune response to the recombinant herpes zoster vaccine (CHO cell) based on multi-omics has adopted the PK/PD trial design.

### 4 Status of vaccine clinical trials in China

**Figure 3:**
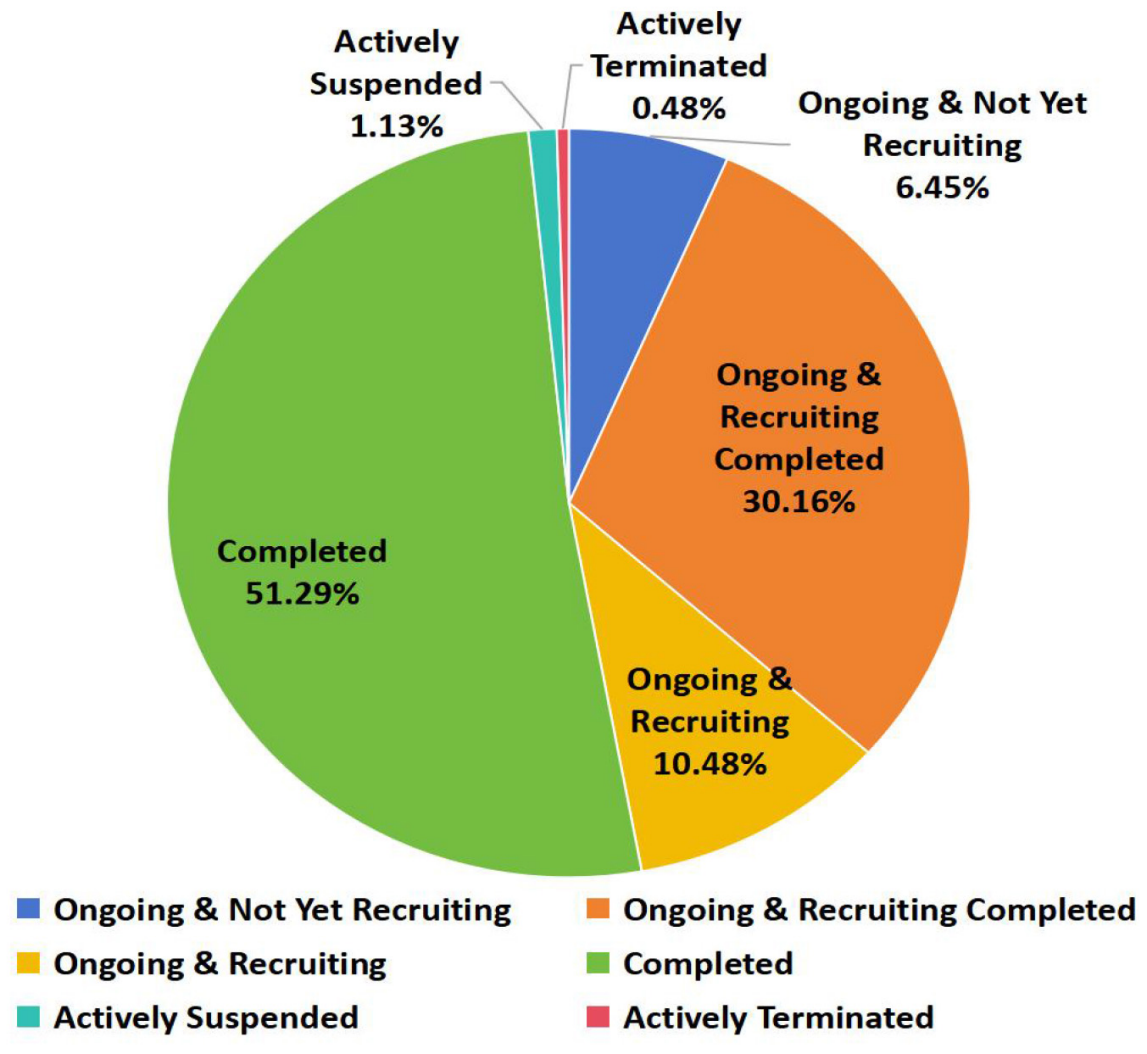
Status of vaccine clinical trials in China.

Figure 3 shows the trial status of vaccine clinical trials in China. Over half of the clinical trials for Chinese vaccines (318 trials) have been completed, with ongoing projects accounting for 47.09% of the total (292 trials) (completed recruitment: 187 trials; ongoing recruitment: 65 trials; not yet recruited: 40 trials). The proportion of trials in abnormal status (voluntary suspension: 7 trials; voluntary termination: 3 trials) remained scarce, totaling only 1.61%.

### 5 Types of vaccines in Chinese-registered clinical trials

Table 2 summarizes the 12 vaccine categories each accounting for ≥2% of all trials, collectively representing 516 (83.2%) studies. The remaining 104 trials (16.8%) involved 35 vaccine types individually contributing < 2%. These included: AC-group meningococcal and Hib combined vaccine (9, 1.5%); HIV vaccine (8, 1.3%); mumps vaccine (8, 1.3%); respiratory syncytial virus vaccine (7, 1.1%); therapeutic BCG (7, 1.1%); norovirus vaccine (6, 1.0%); tetanus vaccine (5, 0.8%); recombinant Staphylococcus aureus vaccine (4, 0.6%); and Shigella bivalent conjugate, tuberculosis, measles–mumps–rubella, plague, Japanese encephalitis, and therapeutic HBV DNA vaccines (3 each, 0.5%). Additionally, bivalent enterovirus inactivated, rubella, cholera, paratyphoid A conjugate, hepatitis A, hepatitis E, DTaP–IPV, and EGF-CRM197 therapeutic vaccines were each investigated in two trials (0.3%). Finally, single trials (0.2%) were registered for BLP25 liposome, HPV-16/18 L1 VLP AS05, NMM therapeutic DNA, RSVPreF3-OA, SVN53-67/M58-KLH, measles–rubella, tick-borne encephalitis, Vi-polysaccharide typhoid, typhoid–paratyphoid A conjugate, DTaP-IPV-Hib, and recombinant human EGF vaccines.

**Table 2:** Types of vaccines in Chinese-registered clinical trials (*N* = 620).

Vaccine type	*n* (%)
Influenza vaccine	106 (17.1%)
Human papillomavirus vaccine (HPV Vaccine)	83 (13.4%)
Pneumococcal vaccine	61 (9.8%)
Meningococcal vaccine	56 (9.0%)
Rabies vaccine	49 (7.9%)
Varicella—zoster vaccine	27 (4.4%)
Poliomyelitis vaccine	26 (4.2%)
Enterovirus 71 vaccine (EV71 Vaccine)	24 (3.9%)
Varicella attenuated live vaccine	23 (3.7%)
Diphtheria, tetanus, and pertussis combined vaccine (dpt vaccine)	21 (3.4%)
Hepatitis B vaccine	21 (3.4%)
Rotavirus vaccine	19 (3.1%)

### 6 Leading institution categories of vaccine trials registered in China

Table 3 lists the four institution types that individually accounted for ≥0.5% of all trials. The remaining four entries (0.3%) were single-count categories (0.16% each): Maternal and Child Health Care Service Center, Research Institute, Drug Clinical Trial Institution, and Medical University + Centers for Disease Control and Prevention (CDC). The distribution of trial phases varies significantly between hospital-led and CDC-led vaccine clinical trials. For hospital-led trials (*n* = 37), Phase I accounts for the largest proportion at 35.14% (13 trials, identified as the primary phase), followed by Phase II at 27.03% (10 trials), Phase III at 18.92% (7 trials), Phase IV at 13.51% (5 trials), and other types at 5.4% (2 trials). In contrast, CDC-led trials (*n* = 571) show a distinct phase focus: Phase III dominates with 36.78% (210 trials, designated as the primary phase), followed by Phase I at 30.12% (172 trials), Phase IV at 14.36% (82 trials), other types at 10% (57 trials), and Phase II at the lowest proportion of 8.76% (50 trials). A key finding from this phase distribution is that hospitals exhibit a clear focus on early-phase clinical research, with the combined proportion of Phase I and II reaching 62.17%, while CDCs play a dominant role in Phase III trials, reflecting the differentiated functional positioning of the two institution types in vaccine clinical trials.

**Table 3:** Categories of leading institutions for vaccine trials registered in China (*N* = 620).

Institution category	*n* (%)
CDC	572 (92.3%)
Hospital	36 (5.8%)
Hospital + CDC	5 (0.8%)
Medical university	3 (0.5%)

### 7 Provincial distribution of leading institutions

**Figure 4:**
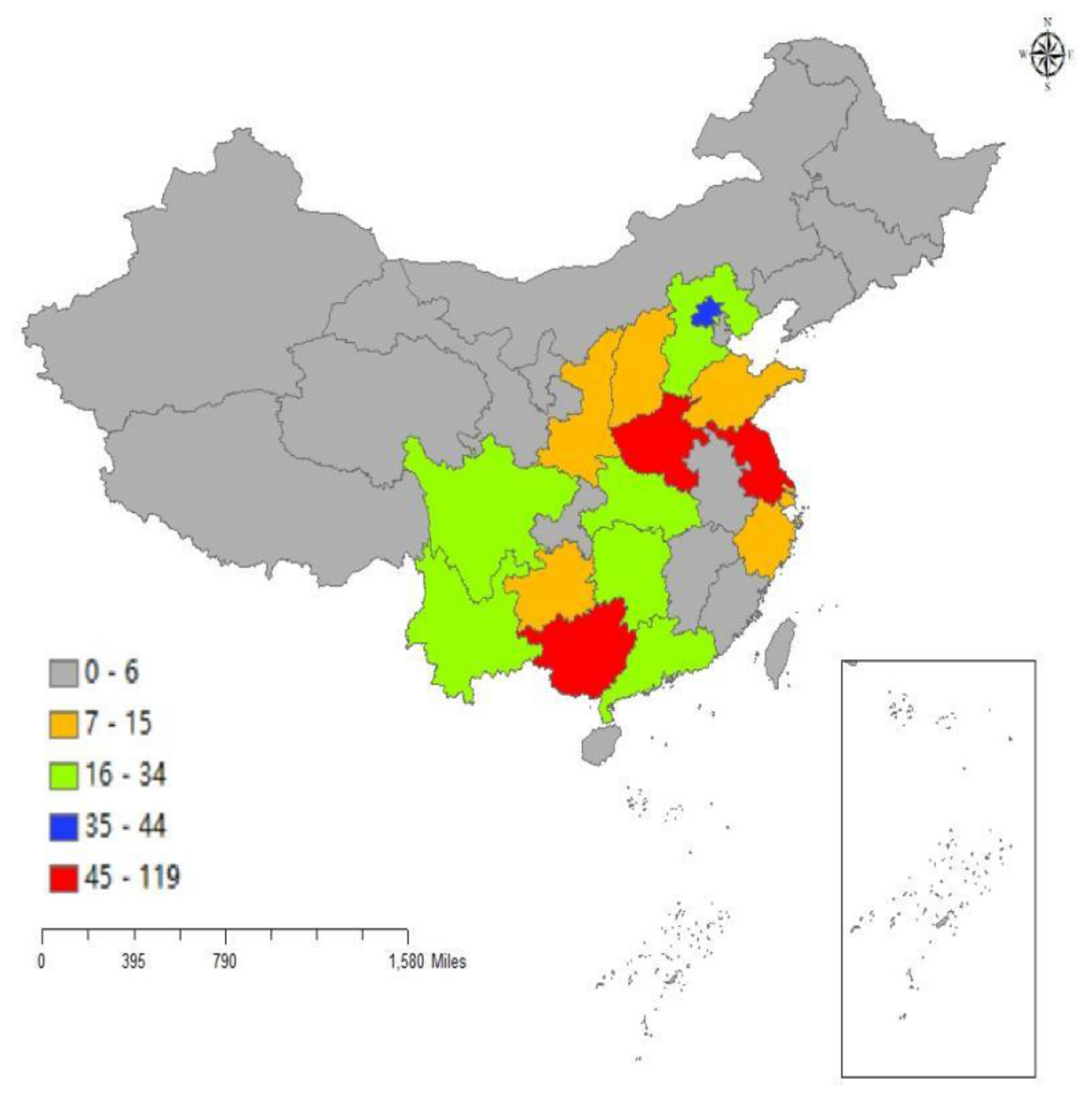
Provincial distribution of lead units in vaccine clinical trials.

The provinces where the leading investigational sites are located exhibited an “east-dense, west-sparse” pattern (Figure 4). Jiangsu ranked first, hosting 119 trials (19.19%), followed by Henan (105 trials, 16.94%) and Guangxi Zhuang Autonomous Region (94 trials, 15.16%); together these three jurisdictions accounted for more than half of the national total (51.29%). Beijing occupied the fourth position (44 trials, 7.10%). Yunnan, Hubei, Hebei, Hunan, and Sichuan each contributed 4%−6%, forming a second-tier cluster. The remaining 13 provinces/autonomous regions/municipalities and the Hong Kong Special Administrative Region each represented < 3%. Shanghai, Chongqing, Inner Mongolia, Fujian, Jilin, Jiangxi and Hong Kong together constituted only 2.90%.

## Discussion

This study analyzed 620 vaccine clinical trials registered on the Platform between 2013 and 2024, revealing key characteristics that reflect China's progress and priorities in vaccine development. First, the dominance of rigorous trial designs—with 84.84% using parallel-group designs, 81.94% randomized designs, and 67.58% double-blind designs. Such designs are critical for minimizing bias in assessing vaccine safety and efficacy, particularly for immunological products where subtle differences in immune response can impact real-world protection. This high adherence to rigorous methods underscores China's commitment to meeting international regulatory expectations, a trend reinforced by post-2019 reforms under the Vaccine Management Law (14). Specifically, after the Law's enactment, significant improvements were observed in two key ethical indicators. The use of DMC increased significantly from 7.79 in 2013–2018 to 17.48% in 2019–2024 (χ^2^ = 11.387, *P* < 0.001), and the proportion of trials providing insurance coverage for participants rose dramatically from 9.52 to 52.96% (χ^2^ = 117.588, *P* < 0.001). These improvements reflect strengthened ethical oversight and participant protection, consistent with the Vaccine Management Law's emphasis on safety monitoring and accountability. For other methodological characteristics, no statistically significant changes were observed between the two periods. However, favorable trends toward more rigorous designs were noted: the proportion of parallel-group trials increased from 82.25 to 86.38%, while single-arm trials declined from 17.75 to 12.85%. Similarly, double-blind design showed an upward trend (from 64.07 to 69.67%), accompanied by a decrease in open-label designs (from 31.17 to 28.28%). Second, phase distribution highlights a focus on late-stage development and early safety validation: Phase III trials (35.75%) and Phase I trials (30.27%) accounted for the largest shares, while Phase II (9.66%) and Phase IV (14.65%) trials were underrepresented. This pattern likely reflects two policy-driven priorities: accelerating market access for vaccines targeting public health needs (via Phase III trials) and mitigating safety risks for novel antigens (via Phase I trials). China's vaccine products have passed the prequalification of the World Health Organization, not only ensuring disease prevention and control domestically, but also meeting the needs of international public health (6). Through the application of platform technology, China accelerated the development and approval process of vaccines, optimized the regulatory procedures, and enhanced the global public health response capacity (15). Third, vaccine type distribution mirrors China's public health priorities: influenza (17.1%), HPV (13.4%), and pneumococcal vaccines (9.8%) were the most studied. Notably, the 2021 and 2023 peaks in trial registrations coincide with investments in respiratory pathogens (e.g., influenza) R&D, demonstrating responsiveness to emerging health threats. Influenza vaccines address seasonal infectious disease burdens, while HPV vaccines target cervical cancer—a leading cause of female mortality in China—aligning with the National Immunization Program's goals.

The dominance of Centers for Disease Control and Prevention (CDCs) as leading institutions (92.26%, 572/620) reflects a distinctive feature of China's vaccine clinical trial landscape. This concentration can be attributed to multiple factors. Under the current regulatory framework, disease prevention, and control institutions qualified to conduct vaccine trials must be at the provincial level or above, as stipulated in the Measures for the Administration of Drug Clinical Trial Institutions (16). In recent years, provincial CDCs across China have been actively advancing the construction of vaccine clinical trial sites, strengthening facility configuration, personnel training, and Good Clinical Practice (GCP) compliance to meet requirements. This ongoing capacity-building effort provides the necessary infrastructure for CDCs to conduct large-scale population-based trials. Although the Vaccine Administration Law permits both tertiary hospitals and provincial CDCs to conduct vaccine trials, hospitals accounted for only 5.81% (36/620) of the trials and were predominantly involved in early-phase studies (Phase I/II). This suggests a functional specialization: hospitals focus on mechanism-of-proof and safety exploration in smaller, tightly controlled settings, whereas CDCs are better equipped for the large-scale, population-based implementation required in Phase III efficacy trials. Strengthening the capacity of hospitals to participate in later-phase trials, possibly through collaborative networks with CDCs, could enhance the overall resilience and diversity of the research system.

The geographic distribution of vaccine trials exhibits a pronounced “east-dense, west-sparse” pattern, with Jiangsu (19.19%, 119/620), Henan (16.94%, 105/620), and Guangxi (15.16%, 94/620) collectively hosting over half (51.29%) of all trials. This clustering reflects regional disparities in research infrastructure, investigator expertise, and local health priorities. Jiangsu's top ranking aligns with its strong pharmaceutical industry base. As the site of the country's only pilot cluster for new vaccines and specific diagnostic reagents, the China Medical City in Taizhou has established a 46,000 m^2^ Vaccine Engineering Center—the largest of its kind in the province—providing integrated services including laboratory R&D, pilot production, testing, and clinical evaluation. By 2025, the region had gathered over 10 human vaccine enterprises, with five obtaining human vaccine production licenses (accounting for 5/9 of the province's total and 1/9 of the national total), and held 16 clinical trial approval documents (17).

Notably, the proportional contribution of traditional hubs such as Beijing and Jiangsu has declined over the study period. Based on the registration data, Beijing's share fell from 22.73 (10/44) in 2013 to 1.49% (1/67) in 2024, while Jiangsu's share dropped from 29.55 (13/44) to 11.94% (8/67) over the same period. In contrast, provinces in central and western China have seen steady growth: Yunnan increased from 2.27 (1/44) in 2013 to 7.46% (5/67) in 2024; Hubei from 2.27 (1/44) to 8.96% (6/67); and Sichuan from 0 (0/44) to 5.97% (4/67). This shift suggests a gradual decentralization of vaccine trial capacity, driven by both national policies aimed at balancing medical research resources and the practical need to access geographically diverse populations. Encouragingly, this trend enhances the generalizability of trial results and fosters regional development of research expertise. Continued investment in training, infrastructure, and regulatory support in under-represented provinces will be crucial to sustain this positive trajectory.

### Trend of trial quantity changes of different vaccine types from 2013 to 2024

Over the past decade, the landscape of vaccine clinical trials in China has exhibited distinct dynamic changes. Influenza vaccines and human papillomavirus (HPV) vaccines have maintained a core position for a long time: influenza vaccines accounted for 15%−28% of total trials annually, peaking at 13 trials in 2019 with an average annual activity of 10.7 trials in the past 3 years (i.e., 2022–2024); HPV vaccines had a share of 9%−25%, reaching 16 trials in 2021 and sustaining an average annual activity of 8.3 trials in the past 3 years (i.e., 2022–2024). Pneumococcal vaccines and herpes zoster vaccines have shown strong expansion: pneumococcal vaccines increased from 3 trials in 2013 to 12 in 2023, with their proportion rising from 8.8 to 20.7%; herpes zoster vaccines, first emerging in 2015, hit eight trials in 2024 and saw their share jump from 4.8 to 15.1%. In contrast, vaccines such as meningococcal vaccines, rabies vaccines, and hepatitis B vaccines have experienced declining activity—hepatitis B vaccines, for example, dropped from a 2013 peak of 7 trials to an average of just one trial per year in the past 3 years (i.e., 2022–2024). Enterovirus 71 vaccines and poliovirus vaccines faded after specific active periods: the former was active during 2016–2019 (peaking at eight trials in 2019) and the latter during 2016–2018.

**Figure 5:**
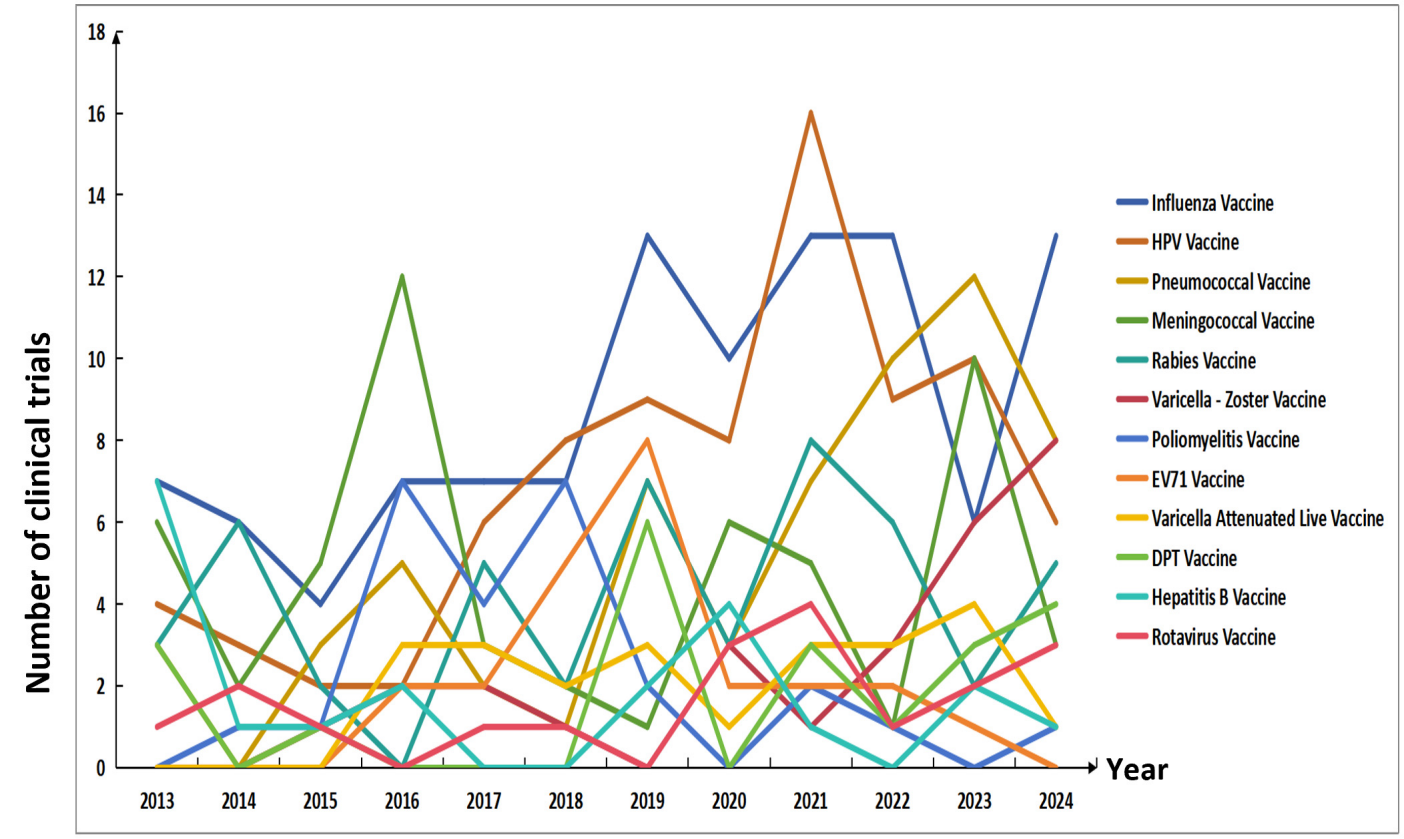
Trend of trial quantity changes of different vaccine types from 2013 to 2024.

This evolutionary pattern reflects two key directions in China's vaccine R&D. On one hand, the number of vaccine types involved in clinical trials increased from 15 in 2013 to 20 in 2023, with the rapid emergence of novel vaccines such as herpes zoster vaccines and rotavirus vaccines, highlighting a prominent diversification trend. On the other hand, the research focus has shifted from traditional areas (e.g., hepatitis B vaccines, poliovirus vaccines) to high-demand fields, including consistently active respiratory vaccines (influenza vaccines, pneumococcal vaccines) and significant cancer-related vaccines (HPV vaccines). Between 2022 and 2024, pneumococcal vaccines (10–12 trials/year) and herpes zoster vaccines (6–8 trials/year) continued their growth momentum, while influenza vaccines (6–13 trials/year) and HPV vaccines (6–10 trials/year) maintained their dominant position, collectively stabilizing the overall scale of clinical trials after the 2019–2021 peak. The specific situation of the change trend in the number of trials of different vaccine types from 2013 to 2024 is shown in Figure 5.

### Summary of the geographic distribution characteristics of vaccine clinical trials

In terms of the number and proportion of vaccine clinical trials across provinces, Jiangsu Province ranked first with 119 trials and an average annual proportion of 20.3%, but its proportion showed a significant downward trend, dropping from 29.6 in 2013 to 11.9% in 2024. Henan Province (105 trials, 16.5% average annual proportion) and Guangxi Zhuang Autonomous Region (94 trials, 16.4% average annual proportion) ranked second and third, respectively. Among them, Henan Province's proportion was relatively stable with a slight decline, while Guangxi Zhuang Autonomous Region's proportion fluctuated between 13 and 38%, remaining stable overall.

**Figure 6:**
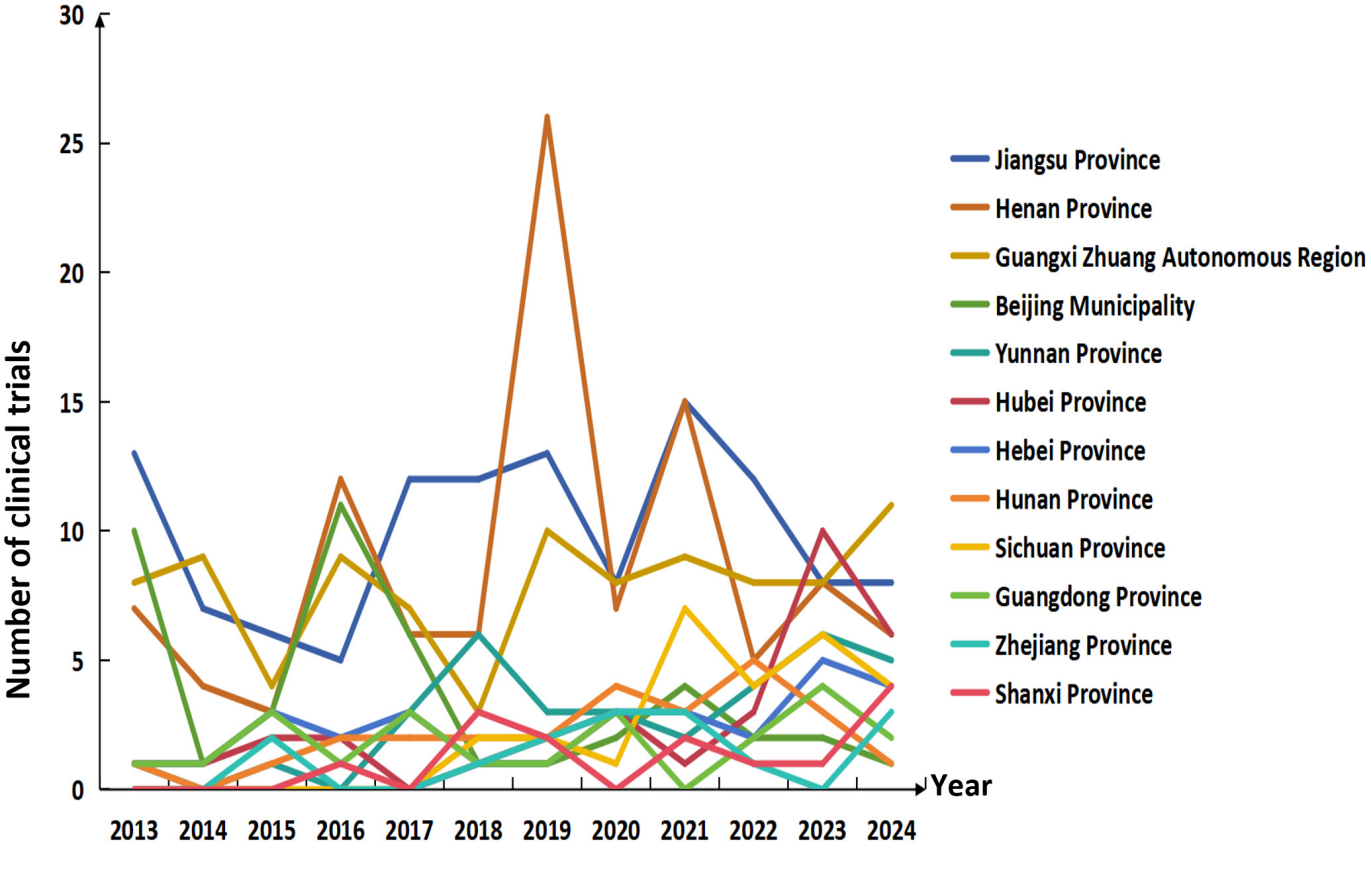
Trends in the number of vaccine clinical trials in major provinces from 2013 to 2024.

Some provinces showed a significant growth trend: the proportions of Yunnan Province and Hubei Province both increased from 2.3 in 2013 to 7.5%, and 9.0% in 2024, respectively; Sichuan Province and Zhejiang Province rose from 0 in 2013 to 6.0%, and 4.5% in 2024, respectively. In contrast, the proportions of Beijing and Jiangsu Province decreased significantly — Beijing's proportion fell from 22.7 in 2013 to 1.5% in 2024, and Jiangsu Province's proportion dropped by 17.6 percentage points. On the whole, the geographic distribution of vaccine clinical trials is shifting from traditional R&D centers such as Beijing and Jiangsu Province to central and western provinces including Yunnan, Hubei, and Sichuan Provinces, as well as Zhejiang Province in eastern China. This trend not only reflects the emerging R&D potential of central and western provinces like Yunnan and Hubei, but also embodies the national strategic orientation of balanced distribution of medical R&D resources, which plays a positive role in promoting the development of regional pharmaceutical industries and enhancing the national vaccine R&D capacity. The specific changes in the geographic distribution characteristics of vaccine clinical trials from 2013 to 2024 are shown in Figure 6.

The United States of America had the highest total number of trials registered during 1999-June 2024 (186,497), followed by China (135,747) and India now with more than Japan (74,031 compared to 65,167) (18). While China holds the second position in terms of the total number of clinical trials, it still lags behind the United States to a certain extent. For instance, a comparative analysis of cancer vaccine trials between China and the USA from 2014 to 2024 reveals that China registered significantly fewer trials (89) compared to the USA (757). The proportion of global trials is markedly lower in China (2.2%) compared to the USA (47.6%) (19). Moreover, the COVID-19 pandemic has underscored the importance of robust vaccine trial infrastructure. A global overview of COVID-19 vaccine development highlighted that South-Southeast Asian countries, including China, carried out more clinical trials proportionally, but these were primarily for mature technologies [21]. To address the aforementioned gaps and challenges, China needs to make targeted efforts across multiple dimensions to comprehensively enhance the quality, internationalization level, and innovation efficiency of clinical trials. On one hand, it should focus on high-value therapeutic areas such as cancer vaccines, increase funding support for original innovative research and development, and reduce follow-up research on mature technologies and repetitive trials. On the other hand, it needs to accelerate the development of global multi-center clinical trials (MRCTs), encourage domestic pharmaceutical companies to take the lead in initiating multi-center trials, and lower the threshold for international collaboration through policy support such as streamlining the approval process for cross-border trials. Additionally, it is necessary to improve clinical trial infrastructure and compliance systems, upgrade digital and intelligent trial management tools to achieve full-process electronic traceability of data, and strengthen the development of professional teams including researchers, monitors, and data managers. Finally, it should continuously optimize policy support and the industrial ecosystem, provide targeted incentives for international multi-center trials and original drug research and development, improve the multi-level payment system for clinical trials, and cultivate high-quality domestic Contract Research Organizations (CROs) to enhance their capabilities in cross-border trial coordination and technical support, thereby reducing the execution costs of international trials.

A potential limitation of this study is that it only included publicly disclosed trials on China's Drug Trial Registration and Information Publication Platform, excluding unpublicized COVID-19 vaccine trials and overseas-conducted Chinese vaccine clinical trials, which may compromise the representativeness and generalizability of the conclusions.

## Data Availability

Publicly available datasets were analyzed in this study. This data can be found here: http://www.chinadrugtrials.org.cn/index.html.
